# Bacterial Immunogenicity Is Critical for the Induction of Regulatory B Cells in Suppressing Inflammatory Immune Responses

**DOI:** 10.3389/fimmu.2019.03093

**Published:** 2020-01-24

**Authors:** Jan Kevin Maerz, Constanze Trostel, Anna Lange, Raphael Parusel, Lena Michaelis, Andrea Schäfer, Hans Yao, Hanna-Christine Löw, Julia-Stefanie Frick

**Affiliations:** Department for Medical Microbiology and Hygiene, Interfacultary Institute for Microbiology and Infection Medicine, University of Tübingen, Tübingen, Germany

**Keywords:** immune regulation, homeostasis, microbiota, regulatory B cells (Bregs), interleukin 10 (IL-10), immunogenicity, inflammatory bowel disease (IBD), Toll-like receptors

## Abstract

B cells fulfill multifaceted functions that influence immune responses during health and disease. In autoimmune diseases, such as inflammatory bowel disease, multiple sclerosis and rheumatoid arthritis, depletion of functional B cells results in an aggravation of disease in humans and respective mouse models. This could be due to a lack of a pivotal B cell subpopulation: regulatory B cells (Bregs). Although Bregs represent only a small proportion of all immune cells, they exhibit critical properties in regulating immune responses, thus contributing to the maintenance of immune homeostasis in healthy individuals. In this study, we report that the induction of Bregs is differentially triggered by the immunogenicity of the host microbiota. In comparative experiments with low immunogenic *Bacteroides vulgatus* and strong immunogenic *Escherichia coli*, we found that the induction and longevity of Bregs depend on strong Toll-like receptor activation mediated by antigens of strong immunogenic commensals. The potent B cell stimulation via *E. coli* led to a pronounced expression of suppressive molecules on the B cell surface and an increased production of anti-inflammatory cytokines like interleukin-10. These bacteria-primed Bregs were capable of efficiently inhibiting the maturation and function of dendritic cells (DCs), preventing the proliferation and polarization of T helper (Th)1 and Th17 cells while simultaneously promoting Th2 cell differentiation *in vitro*. In addition, Bregs facilitated the development of regulatory T cells (Tregs) resulting in a possible feedback cooperation to establish immune homeostasis. Moreover, the colonization of germfree wild type mice with *E. coli* but not *B. vulgatus* significantly reduced intestinal inflammatory processes in dextran sulfate sodium (DSS)-induced colitis associated with an increase induction of immune suppressive Bregs. The quantity of Bregs directly correlated with the severity of inflammation. These findings may provide new insights and therapeutic approaches for B cell-controlled treatments of microbiota-driven autoimmune disease.

## Introduction

The fact that B cells play a critical role during the onset and course of inflammatory processes is indisputable and has been demonstrated in many studies in both mice and humans. However, these studies focused on the production of autoantibodies with B cells being understood as the cause for the development of inflammation, e.g., multiple sclerosis (MS) (1–3). Nevertheless, it is important to discriminate between the versatile functions of B cells. More and more studies have revealed that the depletion of B cells leads to an aggravation of disease in many autoimmune disorders, such as inflammatory bowel disease (IBD) and rheumatoid arthritis (RA) (4). B cells were shown to mediate an anti-inflammatory effect in mice that spontaneously develop chronic colitis, exhibiting more severe disease in the absence of B cells (5–9). Even in experimental autoimmune encephalomyelitis (EAE), an animal model for MS, CD20 antibody-mediated B-cell depletion substantially exacerbated the disease when the treatment was initiated before EAE induction (10–12). This may link the activation of functional B cells with a suppressive effect in inflammation by promoting immune tolerance.

The beneficial influence of B cells during inflammatory processes is primarily attributable to a specific B cell population—regulatory B cells (Bregs) (13). Hitherto, no definitive phenotype with specific markers has been identified for Bregs. Certain phenotypes are characterized and described, but they differ in their expression of surface proteins. However, these phenotypes possess similar functionalities which explain the reason why the definition of Bregs is based on their immune-regulatory and anti-inflammatory capabilities (14). The main features of Bregs include the potent suppression of type 1 T helper (Th) cell differentiation, the inhibition of autoimmune pathogenesis and the maintenance of immune homoeostasis (15). The three most intensely studied Breg subsets are splenic transitional 2 marginal-zone precursor (T2-MZP) cells (CD19^+^CD21^hi^CD23^hi^CD24^hi^), B10 cells (CD19^+^CD5^+^CD1d^+^IL10^+^), and Tim-1^+^ B cells (CD19^+^Tim-1^+^) (16– 21). A common and most important feature of these B cell subsets is the production and secretion of the anti-inflammatory cytokine interleukin 10 (IL-10). IL-10 fulfills regulatory functions by effectively suppressing cell-mediated inflammatory responses, thus restoring Th1/Th2 balance (22–25). IL-10-producing B cells have also been identified in humans (26, 27). The powerful immune downregulation quality of IL-10-producing Bregs has already been shown in various autoimmune diseases, such as EAE, collagen-induced arthritis, lupus and inflammatory bowel disease (9, 28–31). Depending on the type of inflammatory response, there are two major mechanisms by which Bregs suppress inflammation via IL-10: (I) in autoimmune diseases, such as IBD where both innate and adaptive immune responses are involved, Bregs directly dampen the production of proinflammatory cytokines by macrophages (32–34); (II) during inflammatory processes in, for example, EAE and RA, in which an overshooting T cell-mediated response is the driving force for inflammation, Bregs balance Th1/Th2 immune homeostasis (9, 28). Moreover, Bregs shift T cell differentiation to a regulatory phenotype (Tregs) in both mice and humans (19, 35). The influence of regulatory B cells on the induction of Tregs polarization has been verified in B cell-deficient μMT mice and mice harboring a B cell-specific deletion of IL-10. These genotypes revealed a reduction of Treg numbers with a simultaneous increase of proinflammatory Th1 and Th17 cells (19, 36–38).

In addition to the production of IL-10, Bregs express and secrete suppressive molecules and thus possess further mechanisms to regulate immune responses in an IL-10-independent manner: CD73 is a cell-surface enzyme that converts adenosine monophosphate to adenosine with potential immunosuppressive effects (39); PD-L1 (programmed death ligand 1) is an inhibitory costimulatory molecule that restricts T cell differentiation (40–43); FasL (Fas ligand) bound with its receptor induces apoptotic cell death (44–46); GITRL (glucocorticoid-induced tumor necrosis factor receptor-related protein ligand) induces proliferation of Tregs (47); EBI3/IL-35 (Epstein-Barr virus induced gene 3) regulates inflammatory immune responses through induction of Tregs (48–51). In combination, these molecules perfect regulatory B cells to a strong immune-suppressive cell subset.

However, only a few studies have been published that investigate the activation B cells by a direct interaction with viable bacteria *in vitro* and *in vivo*. It has thus far been shown that several bacterial and viral pathogens, as well as parasites, manipulate B cell function directly to modulate host immune responses as part of an immune evasion strategy facilitating their survival and prolonging infection (51–55). Recently published studies highlighted the influence of the resident microbiota on the activation of B cells that modulate intestinal inflammation and promote immune homeostasis (16, 56). The intenseness of B cell activation and differentiation depends on the composition of the host microbiota and the involved resident bacteria which interact and stimulate various immune cells (immunogenicity) (56–58). The immunogenicity of bacteria is pivotal for the strength of provoking an immune response. As demonstrated in recently published studies, the immunogenicity is dependent on the structure of different MAMPs (e.g., LPS) and consequently to the binding affinity to PRRs (59). We thus investigate the immunogenicity-dependent potential of the two model commensals *Bacteroides vulgatus* (weak immunogenic) and *Escherichia coli* (strong immunogenic) to modulate and regulate the immune system of the host via B cells. In this context, we could already show that a weak immunogenic signal provided by *B. vulgatus* is beneficial in genetically predisposed host (deficient for Rag1 or IL-2) in the course of inflammation. In contrast, the administration of strong immunogenic *E. coli* aggravates the disease progression due to the lack of a functional B cell immunity which can restore immune tolerance in a healthy host by counter-regulating the induced pro-inflammatory immune response (59–61).

In this study we demonstrated the following: (I) B cells can be activated directly by commensal members of the host microbiota and, depending on the immunogenic potential of the encountered bacterial species, B cells can mint strong regulatory cell phenotypes to promote immune tolerance; (II) the intensified induction of Bregs by *E. coli* can counter-regulate pro-inflammatory immune responses in a healthy host inherently caused by the same bacteria; (III) this regulation mechanism may serve as a feedback loop to maintain immune homeostasis and even attenuate inflammatory processes in autoimmune disease.

## Materials and Methods

### Bacteria Cultivation

*E. coli* mpk was grown in Luria-Bertani (LB) medium under aerobic conditions at 37°C. *B. vulgatus* mpk was grown in Brain-Heart-Infusion (BHI) medium and anaerobic conditions at 37°C.

### Mice

C57BL/6NCrl mice and C57BL/6-Tg(TcraTcrb)425Cbn/Crl (OT-II) mice were purchased from Charles River Laboratories. Toll-like receptor 2 and 4 double KO mice (*Tlr2*^−/−^, *Tlr4*^−/−^) were provided by Jackson Laboratory. All animals were kept and bred under SPF conditions. For isolation of B cells, T cells and bone marrow, only female mice aged 8–10 weeks were used. Germfree C57BL/6J mice were bred and housed in our own gnotobiotic facility. Animal experiments were reviewed and approved by the responsible institutional review committee and the local authorities.

### Purification and Cultivation of Naïve B Cells and Naïve T Cells

B cells were purified from spleens of WT or TLR2^−/−^ × TLR4^−/−^ mice by magnetic isolation using negative selection with microbeads (Miltenyi Biotec) according to the manufacturer's instruction. The purity of CD19^+^CD43^−^CD4^−^Ter119^−^ B cell population was >95%. B cells were cultured in complete medium (RPMI1640 supplemented with 10% FCS, 50 μM 2-mercaptoethanol, 25 mM Hepes, 1% non-essential amino-acids, 1% sodium pyruvate and 1% penicillin/streptomycin) at a density of 1 × 10^6^ cells/mL in flat-bottom plates for subsequent stimulation experiments. The purity of naïve CD4^+^CD44^−^CD8a^−^CD11b^−^CD11c^−^CD19^−^CD25^−^CD45R^−^CD49b^−^CD105^−^MHCII^−^Ter-119^−^TCRγ/δ^−^ T cell population was >94%. After purification, T cells were directly used for co-culture experiments. For proliferation assays, isolated splenic B cells or T cells were adjusted to a concentration of 10^6^ cells/mL in PBS/1% FCS, resuspended in 10 μM CFSE and incubated at 37°C for 20 min.

### Cultivation of Bone Marrow-Derived Dendritic Cells (BMDCs)

Bone marrow cells were isolated from femurs and tibias of WT mice and cultivated for differentiation in GM-CSF supplemented media as described previously (62). Cells were supplemented with fresh DC media on days 3 and 5. Seven days after isolation, the resulting CD11c^+^ bone marrow-derived dendritic cells (BMDCs) were harvested and used for co-culture stimulation experiments.

### Stimulation of Naïve B Cells and Bone Marrow-Derived Dendritic Cells

1 × 10^6^/mL splenic B cells or BMDCs were stimulated with PBS, *B. vulgatus* mpk or *E. coli* mpk at a Multiplicity of infection (MOI) of 1 at 37°C (“-stimulated B cells/BMDCs”). One μL/mL gentamicin was added to prevent bacterial overgrowth. According to the experimental setting, cells were harvested following stimulation at different time points.

### Co-culture Experiments

For B-T cell co-culture experiments B cells served as APCs and were stimulated for 24 h with commensals as described previously (“-primed B cells”). Prior to co-cultivation with naïve CFSE-labeled OT-II CD4^+^ T cells, B cells were incubated with 10 μg/mL Ova-Peptide (ISQAVHAAHAEINEAGR, EMC) for 2 h at 37°C. B cells were washed and supernatant was exchanged with fresh media (“-pulsed B cells”). Primed and pulsed B cells and naïve T cells were co-cultured at different ratios for 72 h at 37°C and 100 ng/mL purified anti-mouse IL-10 antibody (Clone: JES5-2A5, BioLegend) was added to certain samples.

For CD11c^+^ dendritic cells maturation assay, naïve B cells and differentiated immature BMDCs were simultaneously stimulated with bacteria at MOI 1 and co-cultured at a ratio of 5:1 (B cells/BMDCs). To show the effect of indirect cell-cell interaction, naïve B cells and BMDCs were additionally co-cultured in Transwells (pore-size 0.4 μm) and stimulated with bacteria at MOI 1. To mimic the influence of soluble IL-10 produced by B cells on the maturation of BMDCs, 10 μg/mL recombinant mouse IL-10 (BioLegend) were added to BMDC mono-cultures.

### DSS-Induced Colitis

Germfree (GF) C57BL/6J mice were colonized for 7 days with 1 × 10^8^ *B. vulgatus* or *E. coli* per mL sterile drinking water. After day 8, mice received sterile drinking water without bacteria. Colonization was checked weekly by collecting fresh feces and determining CFU on selective growth agars (Supplementary Figure 1). After 4 weeks of colonization, mice were administered 2% DSS (molecular weight 36–50 kDa; MP Biomedicals, Santa Ana, CA) in their drinking water for 7 days, followed by regular drinking water for 2 days. The disease activity index (DAI) was determined daily by assessment of body weight, stool consistency, and detection of rectal bleeding. On day 9, mice were sacrificed. Colon, spleen and mesenteric lymph nodes (mLN) were removed and cleaned for histological analysis and cell isolation. For the latter, colons were finely minced and incubated in HBSS containing 5 mM EDTA at 37°C for 15 min in motion for 2 cycles. Then, tissue was digested in RPMI 1640 containing 0.4 mg/ml collagenase D (Roche) and 0.01 mg/ml DNase I (Roche) for 20 min at 37°C on a shaking platform. After collagenase digestion, mononuclear cells were collected by centrifugation at 400 g for 5 min. In addition, cells of mesenteric lymph nodes (mLN) and spleen were extracted by gently disrupting the tissue with a sterile syringe plunger and passing through a nylon cell strainer (40-μm mesh) with PBS containing 1% FBS.

Viable cells were counted and cultured for 4 h with 2 μl/mL Leukocyte Activation Cocktail with Brefeldin A (BD Bioscience). After incubation, cells were prepared for flow cytometrical analysis.

### Flow Cytometrical Analysis (FCM)

Harvested cells were washed and FC-receptors were blocked to avoid non-specific binding. Cell death was measured by LIVE/DEAD Cell Viability kit (ThermoFisher) staining for 20 min at 4°C. Intracellular staining was performed using Cytofix/Cytoperm (BD Biosciences) according to the manufacturer's description. For intracellular and cell-surface staining, cells were labeled for 25 min at 4°C with specific fluorophore-conjugated antibodies, washed, and resuspended in staining buffer (PBS/1% FCS). Multi-color FCM analyses were performed on a FACS LSRII (BD Biosciences). For compensation, fluorescence minus one (FMO) samples served as controls. Doublets were excluded using FSC-A/FSC-H gating. Data was analyzed using FlowJo software (Tree Star, Ashland, OR).

Antibodies: CD11c-APC, CD19-BV421, CD1d-AF647, CD21/CD35-BV605, CD23-BB515, CD24 AF700, CD365(TIM-1)-PE, CD4-APC, CD4-Bv605, CD40-APC, CD40-Bv421, CD45R(B220)-FITC, CD5-Bv605, CD80-PerCP-Cy5.5, CD86-PE-Cy7, CD178(Fas-L)-PE, CD274(PD1-L)-PE, CD73-PE, CD210(IL10R)-PE, FoxP3-AF647, I-A/I-E(MHC-II)-FITC, IFNγ-PE-Cy7, IgM-APC, IL10-PE, IL17A-APC-Cy7, IL4-PE, Vβ5.1,5.2-Bv605, GITR-L-PE, Ebi3-PE. All fluorophore-coupled antibodies were purchased from BD Biosciences.

### Cytokine Analysis by ELISA

For determination of TNF and IL-10 concentrations in cell culture supernatants, ELISA Kits purchased from BD Bioscience were used according to the manufacturer's instructions.

### Statistics

Data have been tested for normality using the Shapiro-Wilk normality test. Statistical analyses were then performed via unpaired student's *t*-test or ANOVA for data with Gaussian distribution and via Mann-Whitney or Kruskal-Wallis test for non-parametric statistics. Correlation analyses were performed via Spearman's rank correlation coefficient (*r*). Statistical significance: ^*^p < 0.05, ^**^p < 0.01, ^***^p < 0.001, ^****^p < 0.0001. Error bars represent ± standard deviation (SD).

## Results

### B Cell Activation and Maturation via Strong Immunogenic *E. coli* Are TLR-Dependent

B cells are mainly associated with the production of antibodies against invaders during adaptive immune responses (4). Activation and proliferation of B cells are achieved by either T cell- dependent or -independent interaction, whereas the latter is coordinated by antigen-recognition via membrane-bound immunoglobulin on the B-cell surface, known as B-cell receptor (BCR) (63). However, B cells express additional receptors for the specific and initial recognition of microbial antigens: Toll-like receptors (TLRs) (64). These surface-bound molecules play a key role in the identification of microbe-associated molecular patterns (MAMPs), such as LPS (via TLR4) or PGN (TLR2) by professional antigen-presenting cells (65).

In order to show that B cells are activated and differentiated upon the MAMP recognition via TLRs and that the activation level is dependent on the immunogenicity of encountered microbes, B cells isolated from wild type (WT) and TLR2^−/−^ × TLR4^−/−^ (KO) mice were stimulated with two different immunogenic bacteria: *B. vulgatus* mpk and *E. coli* mpk (66–69).

**Figure 1:**
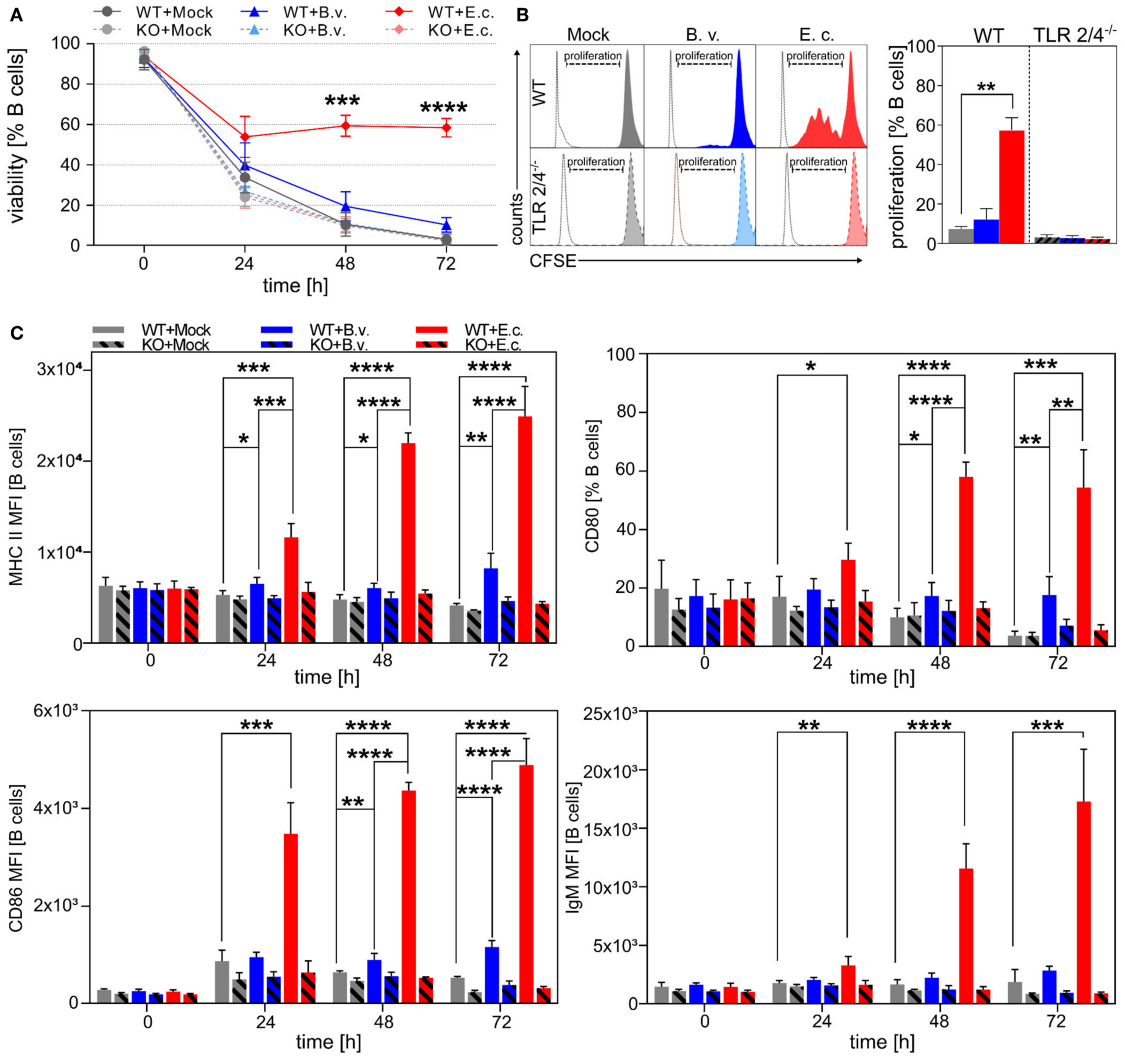
Toll-like receptor-dependent activation of naïve B cells by *E. coli*. Naïve B cells were isolated from spleens of WT or TLR2^−/−^ × TLR4^−/−^ (KO) mice via magnetic cell sorting. B cells were stimulated with *B. vulgatus (B.v.)* or *E. coli (E.c.)* at MOI 1 and PBS as control (Mock) for various time points (0, 24, 48, and 72 h). After incubation, cells were blocked for non-specific binding of antibodies, permeabilized for intracellular antibody staining and fixed prior flow cytometric analysis. **(A)** Survival of CD19^+^ B cells was assessed by staining with life-dead dye. **(B)** B cell proliferation of CD19^+^ B cells was analyzed via CFSE cell labeling. Left panel shows exemplary histograms for each stimulus (colored peaks) including unstained control (white peak). **(C)** Surface expression of IgM, MHC-II, CD80, and CD86 was measured to determine B cell activation after bacterial stimulation (*n* = 3–4). *p < 0.05, **p < 0.01, ***p < 0.001, ****p < 0.0001.

Survival of CD19^+^ B cells was investigated *in vitro* after 0, 24, 48, and 72 h of stimulation with *B. vulgatus* or *E. coli*. The viability of B cells decreased significantly 24 h after stimulation with PBS (Mock) or *B. vulgatus* in comparison to *E. coli*-stimulated B cells, indicating that the survival of B cells is strongly dependent on a potent stimulus with strong immunogenic *E. coli* (Figure 1A). In addition, no significant differences between *E. coli, B. vulgatus* or PBS stimulated TLR2^−/−^ × TLR4^−/−^ B cells were observable, indicating that the activation of naïve B cells depends on interaction of bacterial MAMPs with the corresponding TLRs. This is in line with the analyzed B cell proliferation in response to bacterial encounter after 72 h: a robust bacterial interaction with TLR2 or TLR4 on the surface of B cells is critical for the induction of B cell proliferation (Figure 1B). Stimulation with *E. coli* results in an increased proliferation of naïve B cells expressing TLR2 and TLR4*. E. coli* and *B. vulgatus* failed to activate naïve TLR2^−/−^ × TLR4^−/−^ B cells, resulting in a lack of proliferation. Additionally, maturation and activation markers characteristic for APCs on the surface of splenic B cells were measured after stimulation with bacteria. As shown in Figure 1C, the production and expression of MHC-class-II, CD80, CD86, and IgM were strongly upregulated in response to *E. coli* and increased distinctly over time. In contrast, *B. vulgatus*-challenged B cells expressed only moderate levels of cell activation markers with a constant surface protein expression during 72 h of cultivation. In line with B cell survival and proliferation, the activation of B cells was shown to be TLR2/4 dependent, as TLR2^−/−^ × TLR4^−/−^ splenic B cells did not express significant levels of activation markers, regardless of the bacterial stimulus.

### Strong Immunogenic *E. coli* Induces Differentiation of Regulatory B Cell Phenotypes

**Figure 2:**
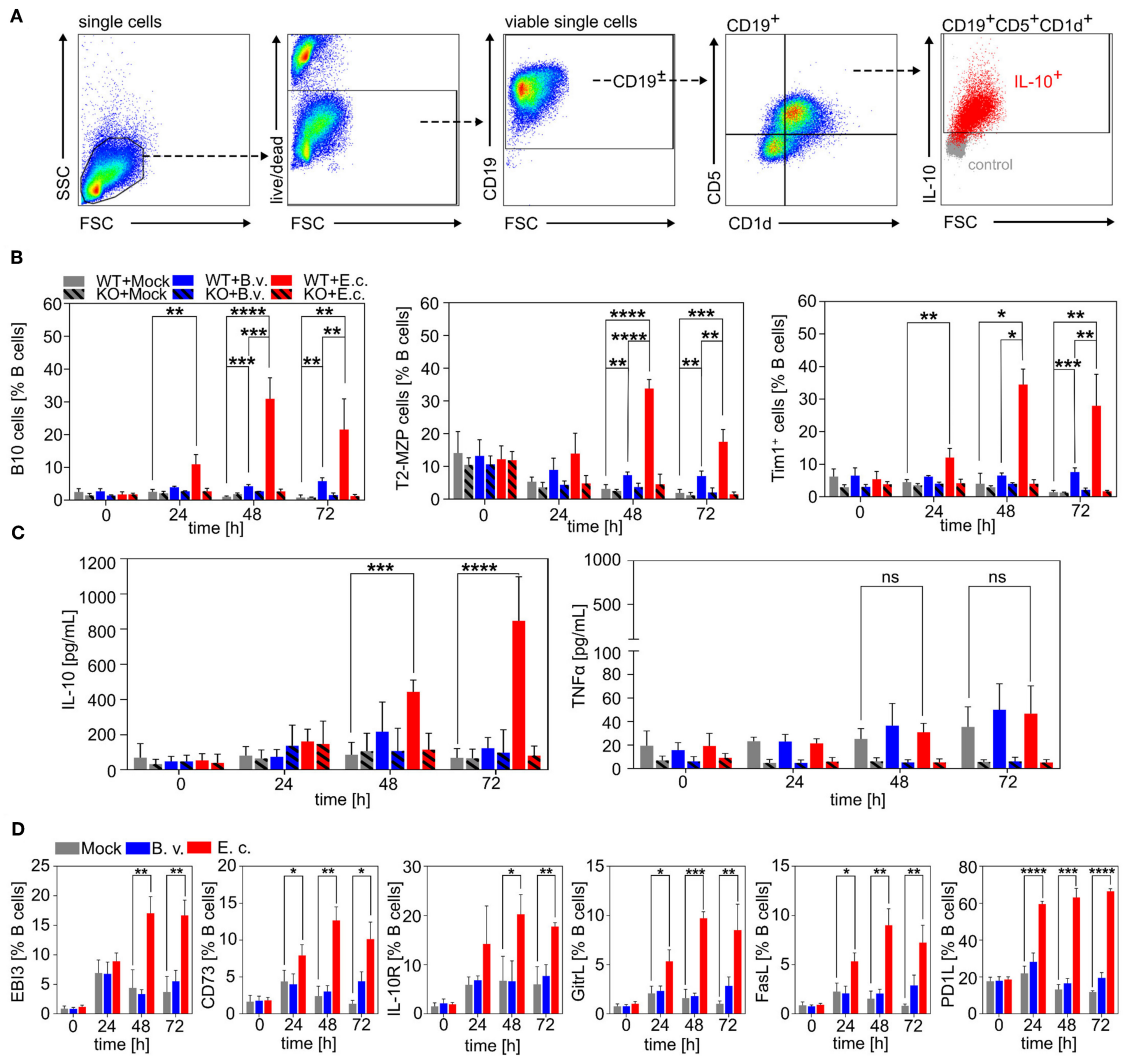
Induction of regulatory B cell phenotypes via *E. coli*. Naïve B cells were isolated from spleens of WT or TLR2^−/−^ × TLR4^−/−^ (KO) mice via magnetic cell sorting. B cells were stimulated with *B. vulgatus (B.v.)* or *E. coli (E.c.)* at MOI 1 and PBS as control (Mock) for various time points (0, 24, 48, and 72 h). **(A)** Exemplary flow cytometry gating strategy for CD19^+^CD5^+^CD1d^+^IL10^+^ (B10 cells) of purified and stimulated splenic B cells **(B)** Induction of regulatory B cell phenotypes CD19^+^CD5^+^CD1d^+^IL10^+^ (B10 cells), CD19^+^CD21^hi^CD23^hi^CD24^hi^ (T2-MZP cells) and CD19^+^Tim-1^+^ (Tim-1^+^ B cells) as determined by flow cytometry. **(C)** Concentration of secreted cytokines IL-10 and TNFα by stimulated B cells measured via ELISA. **(D)** Expression of suppressor molecules on B cell surface after bacterial stimulation as determined by flow cytometry (*n* = 3–4). *p < 0.05, **p < 0.01, ***p < 0.001, ****p < 0.0001.

We first demonstrated that B cells can interact directly with MAMPs via TLRs resulting in differently pronounced survival, activation and proliferation of B cells depending on the recognized microbial antigen. We next determined the cell phenotype of B cells stimulated with *E. coli* or *B. vulgatus*. In order to demonstrate that, in a healthy host, immunogenic bacteria are able to counter-regulate a pro-inflammatory immune response, hence contributing to the maintenance of immune homoeostasis, we focused on the differentiation of naïve B cells to regulatory B cells (Bregs). We determined the percentage of the best characterized Breg subsets: B10 cells, T2-MZP cells, and Tim-1^+^ B cells, at several time points after stimulation with strong immunogenic *E. coli* or low immunogenic *B. vulgatus* in comparison to naïve, unstimulated B cells (Figure 2A) (70). As shown in Figure 2B, the induction of the regulatory B cell phenotypes CD19^+^CD5^+^CD1d^+^IL10^+^ (B10 cells) and CD19^+^CD21^hi^CD23^hi^CD24^hi^ (T2-MZP cells) were significantly enhanced after being challenged with *E. coli* in comparison to *B. vulgatus* or PBS stimulation, reaching the maximum proportion after 48–72 h of incubation (Figure 2B; Supplementary Figures 2, 3). Furthermore, *E. coli-*stimulated B cells differentiated to a higher proportion into CD19^+^ Tim-1^+^ cells (Tim-1^+^ B cells), an additional regulatory B cell phenotype compared to PBS and *B. vulgatus* stimulation (Figure 2B, right; Supplementary Figure 4). Tim-1^+^ B cells are found in the spleen and are reported to produce IL-10 and suppress effector CD4^+^ T cells (70). To further characterize the regulatory properties of PBS, *B. vulgatus* or *E. coli*-stimulated B cells, we determined the concentrations of pro- and anti-inflammatory cytokines secreted in cell culture supernatant. The concentrations of secreted IL-10 were significantly increased upon stimulation of B cells with *E. coli* compared to stimulation with PBS or *B. vulgatus* (Figure 2C). In contrast, only low levels of the pro-inflammatory cytokine TNFα were detectable (<80 pg/mL) and not different in the various treatments (Figure 2C). Furthermore, we analyzed different suppressor molecules produced by immune cells that are not directly linked to a specific Breg phenotype: CD73, PD-L1, FasL, GITRL, and EBI3. *E. coli*, but not *B. vulgatus* stimulation of naïve splenic wild type B cells led to significantly elevated productions of all analyzed immune suppressive molecules starting after 24 h of stimulation (Figure 2D).

### *E. coli-*Stimulated B Cells Inhibit DC Activation and Maturation

**Figure 3:**
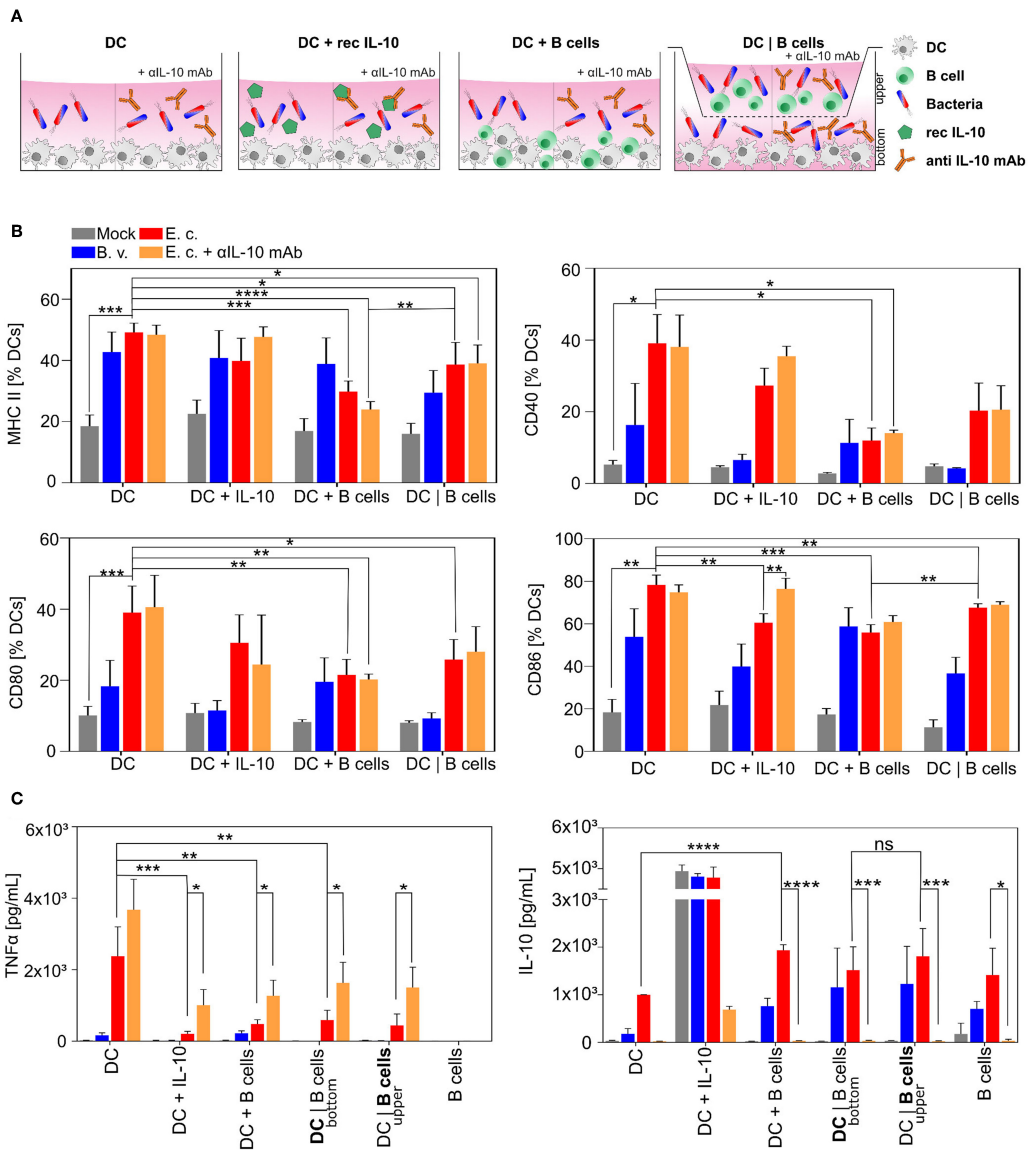
*E. coli*-induced regulatory B cells inhibit maturation of professional APCs *in vitro*. Naïve B cells were isolated from spleens of WT mice and stimulated with *B. vulgatus (B.v.)* or *E. coli (E.c.)* at MOI 1 in co-culture with bone marrow-derived dendritic cells (DC) for 24 h. **(A)** Differentiated bone marrow-derived DCs were cultured alone (DC), with recombinant IL-10 (DC + IL10) or co-cultured with primed B cells without (DC + B cells) or with Transwells (DC|B cells) and challenged with bacteria. **(B)** Maturation of CD11c^+^ APCs was determined through the expression of MHC-II and co-stimulatory markers CD40, CD80, and CD86. **(C)** Concentrations of secreted pro- and anti-inflammatory cytokines TNFα and IL-10 of mono- and co-culture stimulation were determined in the cell culture supernatants. In Transwell stimulations, supernatant of bottom and upper chambers was analyzed (*n* = 4). *p < 0.05, **p < 0.01, ***p < 0.001, ****p < 0.0001.

In the next step we wanted to elucidate whether *E. coli-*activated B cells featuring regulatory properties via the intensified production and upregulated expression of suppressive molecules directly influence the function of other immune cells, e.g., dendritic cells (DCs). We primarily focused on the maturation of differentiated CD11c^+^ bone marrow-derived dendritic cells co-cultured with bacteria-stimulated B cells (Figure 3A). Interaction of immature DCs (iDCs) with MAMPs (e.g., Lipopolysaccharide, LPS) results in DC maturation characterized by an enhanced expression of MHC-II, CD40, CD80, and CD86 and increased secretion of TNFα (61, 68, 69, 71). Activated DCs, as professional APCs, are responsible for a robust immune response via antigen-recognition, -processing, and -presentation resulting in a proper activation of T cells (72, 73).

In line with our group's previous work, stimulation of iDCs for 24 h with strong immunogenic *E. coli* led to a fully mature DC phenotype with a strong upregulation of surface-bound MHC-II, CD40, CD80, and CD86 (Figure 3B, DC dataset, red columns) and high levels of secreted TNFα (Figure 3C, upper panel, DC dataset, red columns). In contrast, the stimulation with low immunogenic *B. vulgatus* resulted in a semi-mature DC phenotype with an intermediate expression of T cell activation markers and alleviated production of TNFα (Figures 3B,C, DC dataset, blue columns) (66, 68, 69). To demonstrate the effect of stimulated B cells on DC maturation, we co-cultured iDCs with B cells either in direct contact (DC + B cells) or separated via Transwell membranes (DC | B cells) and stimulated the cells with PBS, *B. vulgatus* or *E. coli*. *E. coli* challenged B cells inhibit DC maturation during stimulation, as indicated by a notably reduced expression of MHC-II, CD40, CD80, and CD86 (Figure 3B, DC + B cells and DC|B cells dataset, red columns) and significantly diminished secretion of the pro-inflammatory cytokine TNFα (Figure 3C, upper panel, DC + B cells and DC|B cells dataset, red columns). This effect was observed in both direct and indirect DC-B cell co-cultures stimulated with *E. coli*, leading us to conclude that soluble factors able to translocate through the Transwell membrane possess the strongest potential for inhibiting DC maturation. However, the maturation was slightly more suppressed in direct interaction with B cells stimulated with *E. coli*.

As IL-10 has been described as an important regulatory factor of Bregs, mainly driving the modulation of immunological functions of other cell types, we mimicked the effect of IL-10 on DC maturation by adding recombinant IL-10 to DC culture (Figures 3B,C, DC + IL10 dataset) (70). We propose that the stimulation of iDCs with *B. vulgatus* or *E. coli* in the presence of recombinant IL-10 led to a reduced maturation of DCs, indicated by the tendency of lower expression of MHC-II, CD40, CD80, and CD86 in comparison to the stimulation without recombinant IL-10 (Figure 3B, DC + IL10 and DC dataset). We support this hypothesis by the detection of significantly lower levels of secreted TNFα (Figure 3C, upper panel, DC + IL10 and DC dataset) (Figure 3C, upper panel, DC + IL10 and DC dataset).

To investigate the effect of IL-10 secretion by *E. coli*-stimulated B cells on DC maturation, we neutralized IL-10 in the DC-B cell co-culture by adding anti-IL-10 (Figures 3B,C, orange columns). The use of anti-IL-10 mAb partially blocks the inhibitory effect of Bregs on DC maturation, resulting in a restored production of pro-inflammatory cytokine TNFα compared to the stimulation of DCs with *E. coli*-stimulated B cells (Figure 3C, upper panel, orange and red columns). Further, we measured the concentration of IL-10 in the cell culture supernatant of all samples and proved the equal distribution of secreted cytokines through the Transwell membranes in the upper and bottom chamber (Figure 3C, lower panel). As expected, and already shown in Figure 2B in single cell cultures, the concentration of IL-10 in co-culture supernatant was highest in *E. coli-*stimulated samples (Figure 3C, lower panel, red columns).

### *E. coli*-Primed B Cells Inhibit T Cell Activation and Induce Treg Differentiation

**Figure 4:**
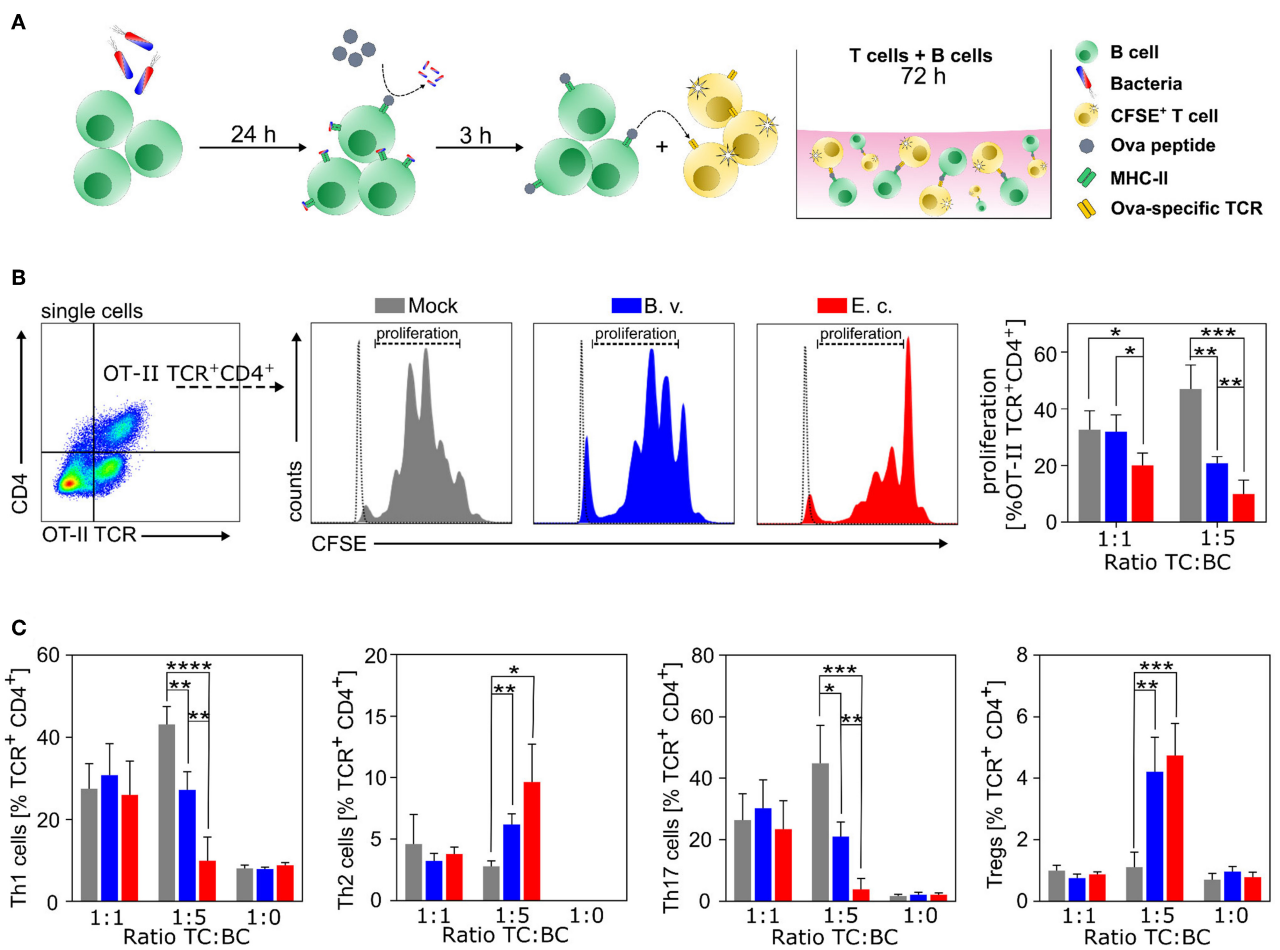
*E. coli*-induced Bregs inhibit T cell proliferation and direct T cell polarization *in vitro*. **(A)** Naïve B cells were isolated from spleens of WT mice and primed with *B. vulgatus (B.v.)* or *E. coli (E.c.)* at MOI 1 for 24 h. T cell proliferation and polarization assays were performed with Ova peptide-specific TCR-expressing naïve T cells in combination with primed and with Ova peptide-pulsed B cells at various ratios. **(B)** Left panel shows exemplary flow cytometry gating strategy. Middle panel shows exemplary histograms for each stimulus (colored peaks) including unstained control (white peak). Percentage of proliferated TCR^+^CD4^+^ T cells was assessed by CFSE staining (right panel). **(C)** Polarization of naïve T cells incubated for 72 h with different stimulated B cell ratios: Th1 (TCR^+^CD4^+^INFγ^+^), Th2 (TCR^+^CD4^+^IL-4^+^), Th17 (TCR^+^CD4^+^IL-17a^+^) cells, and Treg (TCR^+^CD4^+^FoxP3^+^) cells (*n* = 4). *p < 0.05, **p < 0.01, ***p < 0.001, ****p < 0.0001.

As presented above, we were able to show that commensal primed B cells significantly shape DC maturation and consequently influence antigen presentation and the potential to activate T cells. Next, we investigated the direct effect of commensal activated B cells on the proliferation and polarization of T cells without the involvement of professional APCs. We primed WT B cells with PBS, *B. vulgatus* or *E. coli* for 24 h, pulsed the cells with Ova-peptide and co-cultured these B cells at different cell ratios (1:1, 1:5) for 72 h with naïve CFSE-labeled CD4^+^CD44^−^ T cells isolated from OT-II mice, which express an Ova peptide-specific TCR (Figure 4A). The proliferation of naïve CD4^+^ T cells was significantly lower in co-cultures with *E. coli-*primed B cells, with a T cell-B cell ratio of 1:1 in comparison to PBS or *B. vulgatus*-primed B cells (Figure 4B). Increasing the number of antigen-pulsed B cells (ratio T/B 1:5) resulted in a potent reduction of T cell proliferation in both *E. coli-* and *B. vulgatus-*primed cells (Figure 4B). B cells as *bona fide* APCs, usually direct the induction of Th2 responses by suppressing the differentiation of Th1 and Th17 cells and promoting regulatory T-cell expansion (74–76). Th1 (CD4^+^IFNγ^+^), Th2 (CD4^+^IL4^+^), Th17 (CD4^+^IL17A^+^IL4^−^IFNγ^−^) cell, and Treg (CD4^+^FoxP3^+^) differentiation in B-T cell co-culture was analyzed by flow cytometry. PBS-primed naïve B cells appeared to favor the differentiation of naïve T cells to Th1 and Th17 cells, whereas *E. coli*-primed B cells led to a significantly enhanced T cell polarization toward Tregs. In addition, co-culture of *E. coli*-primed B cells with naïve T cells resulted in a polarization of Th2 cells at a T cell-B cell ratio of 1:5 (Figure 4C) and the frequency of Th1 cells significantly decreased with a simultaneous increase of Th2 cells compared to PBS control.

### Induction of Begs by Strong Immunogenic *E. coli* Contributes to the Counter-Regulation of DSS-Induced Inflammation

In the *in vitro* experiments, we could show that B cells, especially regulatory B cells, play a crucial role during the initiation of immune responses and that B cells can directly or indirectly affect the function of other immune cells by their regulatory properties.

**Figure 5:**
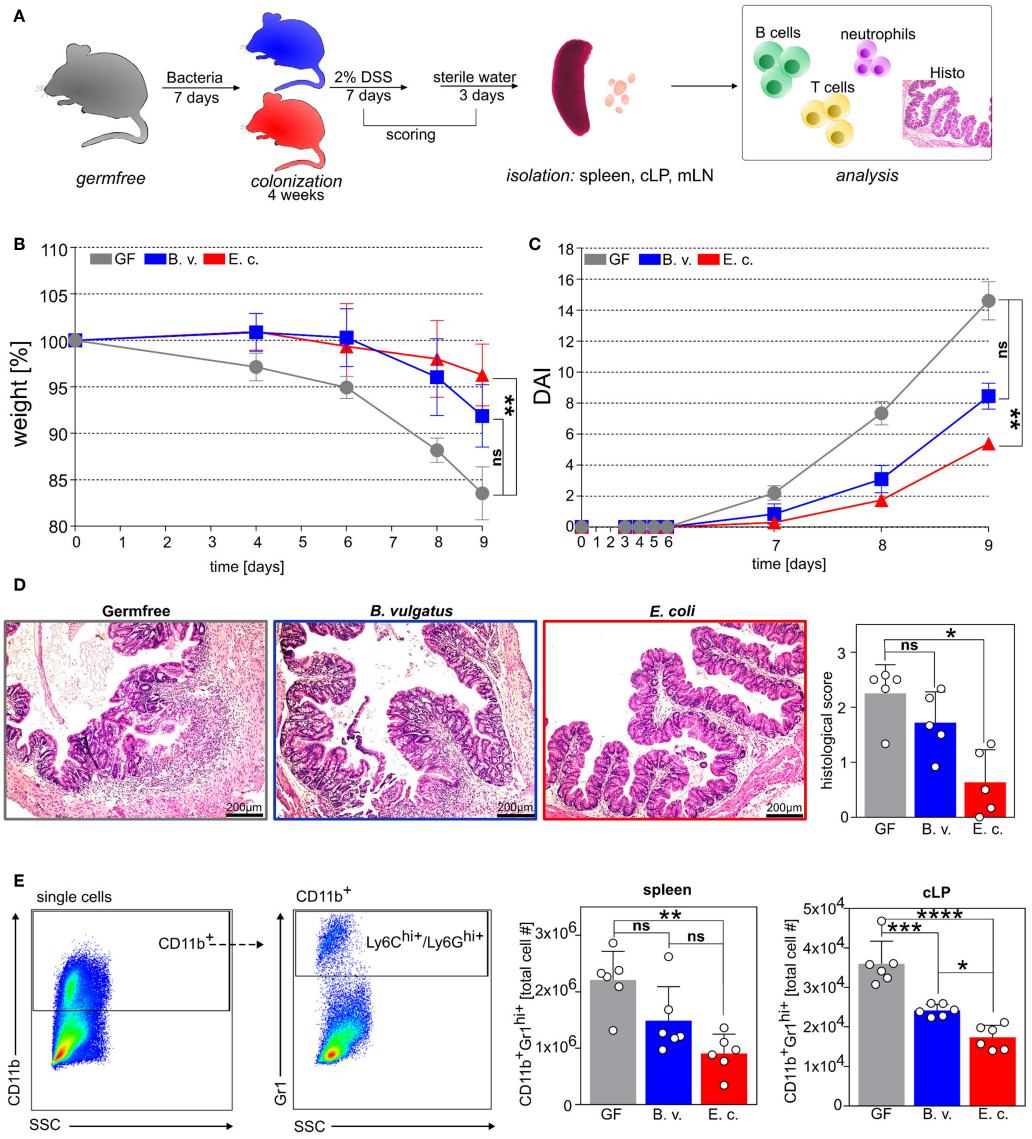
*E. coli* colonization of germfree mice ameliorates DSS-induced colitis. **(A)** Germfree wild type mice and *B. vulgatus-* or *E. coli-*colonized wild type mice were administered with 2% DSS in drinking water for 7 days, followed by regular drinking water for an additional 2 days. **(B)** Bodyweight changes and **(C)** disease activity index (DAI). **(D)** Representative colon histological sections stained with hematoxylin and eosin (H&E) and histological score. **(E)** Exemplary flow cytometry gating strategy and analysis of absolute infiltrated CD11b^+^GR1^+^ neutrophils in spleen and colonic lamina propria (cLP) (GF: *n* = 5; B.v/E.c: *n* = 5–6). *p < 0.05, **p < 0.01, ***p < 0.001, ****p < 0.0001.

This led us to the hypothesis that in a host having functional B cell immunity, *E. coli* with its immunogenic potential counter-regulates inflammatory processes via the strong induction of regulatory cell populations and can consequently help to maintain immune homeostasis. Therefore, we used the Dextran Sulfate Sodium (DSS)-induced colitis model in gnotobiotic C57BL/6 mice which have a physiological B and T cell response. We colonized germfree WT mice with *B. vulgatus* or *E. coli* for 4 weeks via drinking water (Supplementary Figure 1) and administered 2% DSS for 7 days in order to correlate the bacteria-dependent induction of regulatory B cells in spleen and mesenteric lymph nodes (mLN) with the disease pathology (Figure 5A).

**Figure 6:**
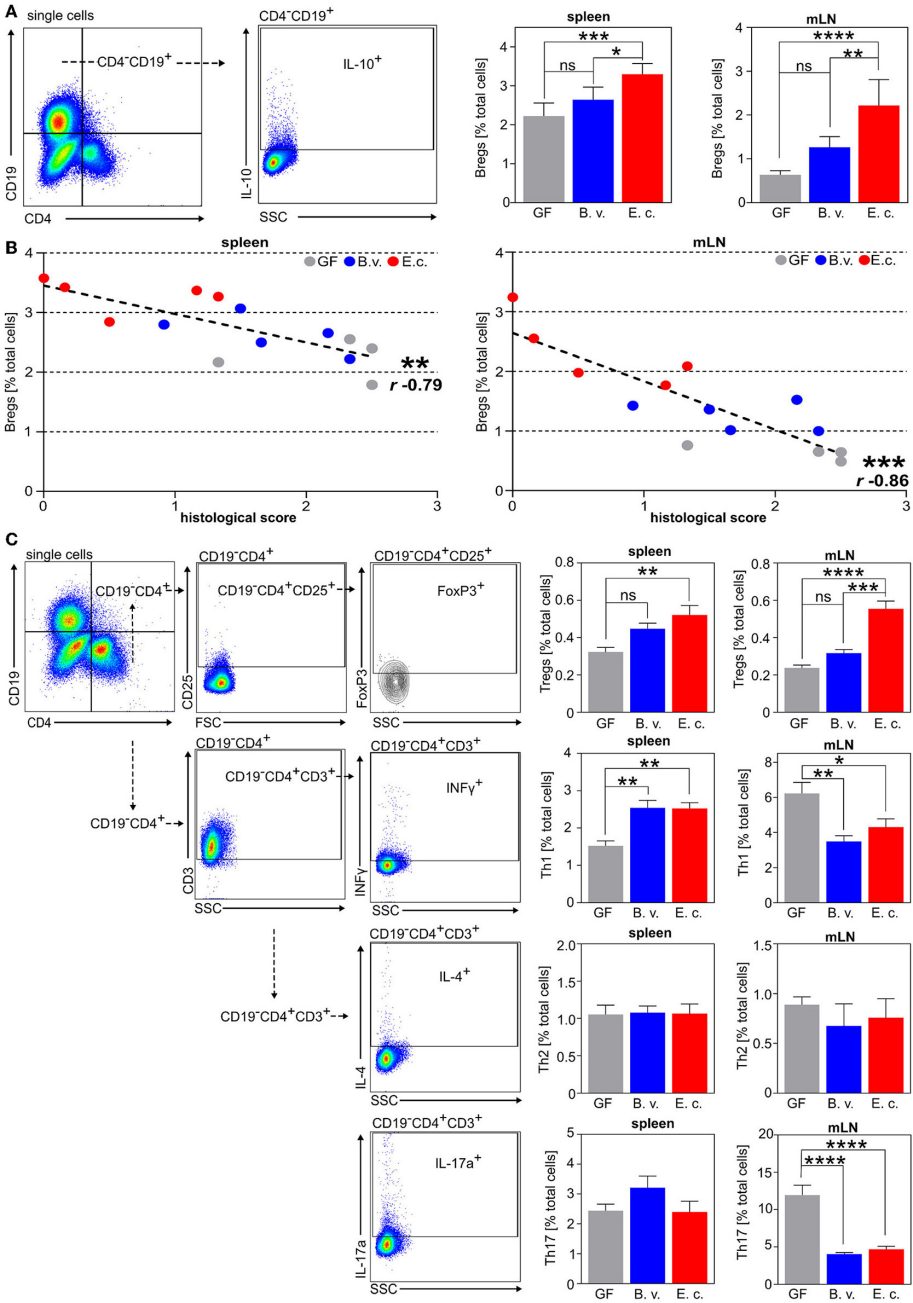
*E. coli* colonization of germfree mice triggers the induction of regulatory B cells accompanied by an increase of Treg cells during DSS-induced colitis. **(A)** Exemplary flow cytometry gating strategy and analysis of CD4^−^CD19^+^IL-10^+^ regulatory B cells (Bregs) in the spleen and mesenteric lymph nodes (mLN). **(B)** Correlation between histology colitis score and percentage of CD4^−^CD19^+^IL-10^+^ Bregs in the spleen (left panel) and mLN (right panel) of colonized and DSS-administered mice (*r* = Spearman's rank correlation coefficient). **(C)** Exemplary flow cytometry gating strategy and analysis of Th1 (CD19^−^CD4^+^INFγ^+^), Th2 (CD19^−^CD4^+^IL-4^+^), Th17 (CD19^−^CD4^+^IL-17a^+^) cells, and Treg cells (CD19^−^CD4^+^CD25^+^FoxP3^+^) in the spleen and mesenteric lymph nodes (mLN) (GF: *n* = 5; B.v/E.c: *n* = 5–6). *p < 0.05, **p < 0.01, ***p < 0.001, ****p < 0.0001.

Colonization of germfree mice with *E. coli*, but not with *B. vulgatus*, resulted in significantly reduced weight loss and a lower disease activity index in comparison to germfree mice in response to DSS-administration (Figures 5B,C). Additionally, the histological score of tissue damage in the colon was significantly attenuated in *E. coli-*colonized mice but not in *B. vulgatus-*associated mice as compared to germfree mice (Figure 5D). Further, the increased absolute numbers of CD11b^+^Gr1^+^ neutrophils in the spleen and the infiltration of these neutrophils in the colonic tissue implied a severe inflammatory state in germfree mice after DSS treatment in comparison to *E. coli*-colonized mice (Figure 5E). Interestingly, the influx of neutrophils was also reduced in *B. vulgatus*-colonized mice as compared to germfree mice. The percentage of CD4^−^CD19^+^IL10^+^ regulatory B cells in the spleen and mLN was significantly increased in *E. coli*-colonized mice in comparison to *B. vulgatus*-colonized and germfree animals before and after DSS-administration (Supplementary Figure 5, Figure 6A). This enhanced Breg induction correlated with a lower histological score, whereas highly inflamed mice showed a minimal activation of regulatory B cells in the spleen and mLN (Figure 6B). In addition, we analyzed the polarization of CD4^+^ T cells in all three DSS-administered groups. No significant differences were observed in the Th2 cell differentiation between the groups (Figure 6C). However, *E. coli-*colonized mice showed a significant increase of regulatory T cells (CD19^−^CD4^+^CD25^+^FoxP3^+^) in the spleen and mLN, whereas the proportion of Th1 (CD19^−^CD3^+^CD4^+^IFNγ^+^) and Th17 (CD19^−^CD3^+^CD4^+^IL-17^+^) cells was significantly reduced in the mLN in comparison to germfree DSS-treated mice (Figure 6C).

In summary, we showed *in vitro* that the activation of naïve B cells and the differentiation of Bregs are highly dependent on the strength of immunogenicity of the interacting commensal bacteria. Moreover, the enhanced induction of Bregs by strong immunogenic *E. coli* in combination with the differentiation of Tregs may contribute to the regulation and even suppression of inflammatory processes *in vivo* as compared to *B. vulgatus*.

## Discussion

In healthy individuals, a complex interaction between the host immune system and commensal microbiota is required to maintain intestinal homeostasis.

Alterations of the gut microbial communities can cause an aberrant and dysregulated immune system resulting in the onset or aggravation of inflammatory bowel diseases, such as Crohn's disease (CD) and ulcerative colitis (UC) (77–79). Furthermore, a disturbed immune system-microbiota balance has also been described in disease pathologies affecting tissues other than those of the intestine (e.g., Grave's disease, multiple sclerosis, type-1 diabetes (T1D), systemic lupus erythematosus, psoriasis, schizophrenia, and autism spectrum disorders) and can be the cause for inflammatory processes during disease development (80–89).

Beside pathogens which are typically acquired from the environment, often provoking acute infections and are the target of immunity, also members of the resident microbiota, found in most healthy hosts, may be the cause for several inflammatory disorders in a genetic or environmental predisposed organism (90–94). The exact mechanisms mediating pathology remain largely unclear, but commensal bacteria are capable of triggering inflammatory disease in immunosufficient rodents by altering barrier function, invading the gut epithelium and stimulating local inflammatory responses (pathobionts) (93, 94).

However, it is conceivable that pathobionts do not actively use specific mechanisms to harm the host under certain circumstances; rather, mechanisms, signaling pathways or cell functions are malfunctioning or are lacking in an immune-compromised host, which pathobionts usually activate in a healthy host to counter-regulate inflammatory processes.

But which immune response mechanisms are consulted by commensals in a healthy host to maintain immune homeostasis? One possible mechanism could be the induction of regulatory B cells.

Most studies investigate the interaction of pathogenic bacteria with B cells during infection. Alternatively, we wanted to demonstrate that the induction of regulatory B cell populations is a mechanism which commensals use to suppress self-caused pro-inflammatory immune responses in a healthy host (54, 95). We also wanted to confirm that Bregs, although present in a minor cell frequency, are an interface in immune-regulation and thus an important player in counter-regulation of inflammatory immune responses.

Primarily, naïve B cells are activated via the help of Th cells which present microbial antigens to the B cell receptor (BCR) followed by the uptake of the antigen through receptor-mediated endocytosis, degradation and presentation of peptide in complex with MHC-class-II molecules on the B cell membrane (96). The recognition of this complex by the corresponding T cell receptor (TCR) of Th cells and the concurrent binding of CD40L with the CD40 receptor on the surface of B cells, as well as the secretion of co-stimulatory cytokines, such as IL-4 and IL-21 by the T cells, promote B cell proliferation, immunoglobulin class switching and somatic hypermutation (T cell-dependent activation) (96, 97). However, we propose a T cell-independent activation and induction of Bregs by commensal bacteria via the direct interaction of MAMPs with TLRs expressed on B cells. Especially the exposure of naïve B cells to LPS and peptidoglycan (PGN), the two most prominent immune-stimulatory bacterial cell components, were responsible and necessary for a potent B cell proliferation and differentiation of Bregs since activation markers like MHC-II, CD80 and CD86 were at lower levels expressed on B cells lacking TLR2 and TLR4 after bacteria encounter (65, 98–101). We could provide evidence for a T cell-independent activation of B cells even without other co-stimulatory factors [e.g., CD40 engagement, CD80/CD86, B cell-activating factor (BAFF), or BCR-TLR crosstalk] that, according to many studies, are pre-requisited to promote an optimal B cell activation (102–105).

Further, we suggest that not only the presence of pattern recognition receptors (PRRs), such as TLRs on the B cell surface is crucial for B cell activation. Additionally, the antigen origin and its immune-stimulatory level might be decisive for the strength of activation and thus the differentiation and survival of Breg subsets.

We could demonstrate that commensal bacteria possess different potentials for directly activating B cells, prolonging their survival and inducing Breg phenotypes as well. In this comparative study of *B. vulgatus* (low immunogenic) and *E. coli* (strong immunogenic), we were able to show that the potent stimulation of naïve B cells with *E. coli* resulted in a strong activation and differentiation of Breg phenotypes characterized by a significantly elevated secretion of anti-inflammatory IL-10 and higher production of suppressive molecules like EBI3, CD73, GITRL, FasL, and PD-L1. In addition, the secretion of the pleiotropic modulatory cytokine IL-6, that exerts either pro-inflammatory or anti-inflammatory properties, was upregulated past stimulation of B cells with *E. coli* whereas only low levels of pro-inflammatory TNFα were detectable (Supplementary Figure 6). IL-6 can directly promote Breg cell differentiation and IL-10 production *in vivo* and therefore could be an important trigger for the proceeding induction of Bregs (16, 106, 107).

Important for all following experiments, we demonstrated in *in vitro* stimulation a functioning direct interaction of commensal bacteria with naïve B cells. Nevertheless, the encounter of naïve B cells with microbial antigens *in vivo* is more complex and subject to many limitations, such as intestinal barrier functions and the translocation of microbial structures to secondary lymphoid organs as with the lymph nodes and the spleen. Furthermore, B cells are not capable of recognizing and taking up microbial components via cell protrusions as dendritic cells perform (108). However, Hudak et al. detected a large number of CD19^+^ B cells in colonic lamina propria cells which were positive for the uptake of PGN of D-amino acid hydroxycoumarin amino-D-alanine (HADA)-labeled bacteria 4 h after oral gavage (109). This study indicated that CD19^+^ B cells act as APC in the lamina propria and hence contribute to the regulation of immune processes by directly recognizing MAMPs.

**Figure 7:**
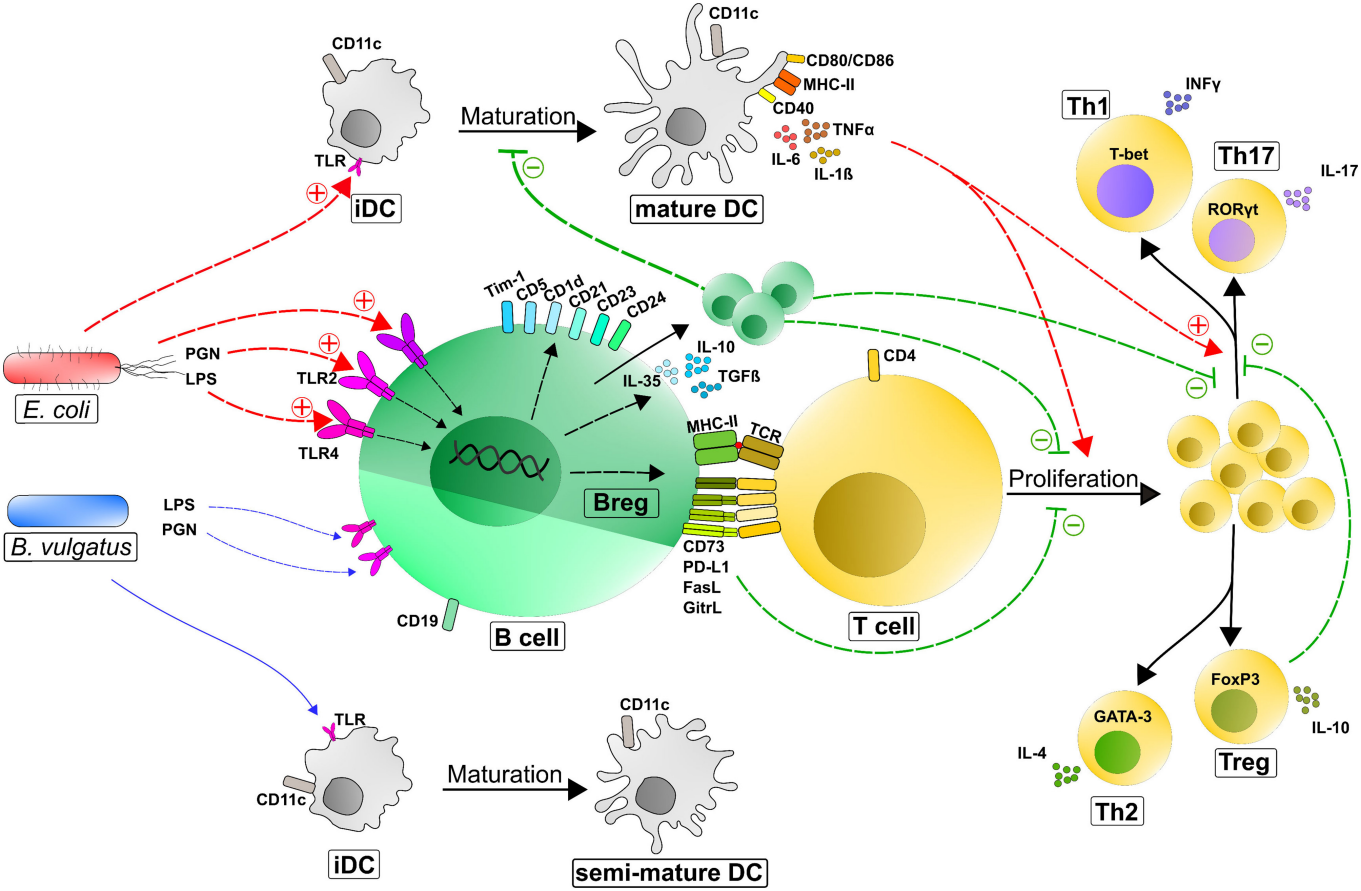
Counter-regulatory mechanisms of Bregs after the induction of immune responses via immunogenic bacteria. Depending on the immunogenicity of a bacterial antigen, APCs (immature DCs and naïve B cells) are activated through the recognition of MAMPs (e.g., LPS or PGN) via Toll-like receptors to different degrees. Potent stimulation of B cells (e.g., by strong immunogenic *E. coli*) induces the differentiation and proliferation of regulatory B cell phenotypes, such as B10 cells (CD19^+^CD5^+^CD1d^+^IL10^+^), T2-MZP cells (CD19^+^CD21^hi^CD23^hi^CD24^hi^), and Tim-1^+^ B cells (CD19^+^ Tim-1^+^) characterized by an upregulated expression and strong secretion of suppressive mediators. Primarily through secreted IL-10, Bregs can inhibit DC and macrophage maturation and function and consequently dampen their antigen presentation capacity to activate and polarize T cells. Moreover, Bregs can regulate T-cell responses by suppressing the proliferation and polarization of effector T cells (in particular TH1 and TH17 cells). These effects are mediated by secreted factors (IL-10, TGFß, and Ebi3/IL-35) and membrane-bound molecules including MHC-II, CD73, PD-L1, FasL, and GitrL at the interface between B cells and T cells (115). Regulatory B cells also crosstalk with Treg cells to promote their expansion and support their function. +, induction/activation; –, inhibition; semi-mature DCs, intermediate expression of DC maturation markers; LPS, lipopolysaccharide; PGN, peptidoglycan; TLR, toll-like receptor; iDC, immature dendritic cell; CD, cluster of differentiation; PD-L1, programmed death ligand 1; FasL, Fas ligand; GitrL, glucocorticoid-induced tumor necrosis factor receptor-related protein ligand; IL–, interleukin–; TCR, T cell receptor; TGFß, transforming growth factor ß; Th, T helper cells; Tim-1, T cell Ig and mucin 1; IFNγ, interferon γ; TNFα, tumor-necrosis factor α; T-bet, T-box transcription factor; RORγt, RAR-related orphan receptor gamma; GATA-3, Trans-acting T-cell-specific transcription factor GATA-3; FoxP3, Forkhead box protein P3.

We determined that the bacteria-B cell interaction with the accompanying induction of regulatory B cells had extensive effects on other immune cells and the overall immune response. We demonstrated this in the interplay between bacteria-induced Bregs and DCs. It is already known that DCs, as professional APCs, have crucial influences on B cell function by capturing unprocessed antigen and transferring this antigen to naïve B cells, resulting in the initiation of antigen-specific antibody response and the provision of B cell isotype class switching (110–112). Here, we represented the effect of activated B cells on DC differentiation, maturation and function. The stimulation of immature DCs (iDCs) with *B. vulgatus* led to a semi-mature, tolerant phenotype characterized by a low expression of MHC-II, CD40, CD80, and CD86 and modest secretion of TNFα and IL-1ß. In contrast, the challenge of iDCs with *E. coli* resulted in a high expression of MHC-II and co-stimulatory markers with increased secretion of pro-inflammatory cytokines (60, 66–69, 113, 114) (Figure 7).

However, in our co-culture experiments, the interaction of DCs with *E. coli*-stimulated B cells significantly inhibited DC maturation, marked by a reduced expression of DC activation and maturation markers and an alleviated secretion of pro-inflammatory cytokines. This suppression of DC maturation was mediated by distinct regulatory properties of *E. coli*-induced Bregs. We could show in Transwell co-cultures that the regulatory functions of Bregs on DC maturation were not solely mediated by the anti-inflammatory cytokine IL-10 since the addition of recombinant IL-10 was not as effective as the complete repertoire of proteins secreted by stimulated B cells. Consequently, additionally secreted molecules, such as EBI3/IL-35 can contribute to the DC maturation inhibition properties of *E. coli*-stimulated B cells in Transwell co-culture experiments (51) (Figure 2D).

Furthermore, maturation of DCs was even more reduced in co-culture stimulation where DCs and *E. coli*-stimulated B cells had direct cell-cell contact. This is in line with our previously findings that the stimulation of B cells with *E. coli* led to an increased upregulation of suppressive molecules like PD-L1, FasL and GITRL on the B cell surface and that these proteins, in combination with secreted cytokines, characterize the strong anti-inflammatory feature of Bregs (47, 51, 116). Moreover, although the expression of T cell activation markers and the production of pro-inflammatory cytokines by DCs were suppressed via *E. coli*-stimulated B cells, the concentration of anti-inflammatory IL-10 in the DC-B cell co-culture was significantly higher than in single cell cultures of DCs and B cells. This led us to the hypothesis that *E. coli*-challenged DCs and B cells may even form a positive feedback loop, resulting in an accumulated anti-inflammatory milieu to regulate overshooting immune responses and dampen inflammatory processes in a healthy host (117, 118).

We further demonstrated that this B cell-driven regulatory mechanism had relevant impacts on the subsequent processes of the adaptive immune response: the activation, proliferation and polarization of T helper cells. Schmidt et al. revealed that Ag-pulsed splenic B cells possess a stronger T cell stimulatory capacity than CD11c^+^ DCs and that activated CD4^+^ T cells favor Th2 polarization *in vitro* (119). Saze et al. investigated T cell activation properties of B cells in more detail. They compared the proliferation of T cells incubated with either naïve B cells or activated B cells and showed that freshly purified B cells co-cultured with separated CD4^+^ T cells augment proliferation of the T cells as well as their cytokine production whereas activated B cells significantly inhibit T cell proliferation (120). By using two commensals featuring different immunogenic properties for B cell stimulation, we could complement and confirm these findings; furthermore, we could expand our understanding of the mechanism behind microbiota-mediated B cell regulation. The stimulation of CFSE-labeled Ova peptide-specific TCR^+^CD4^+^ T cells with *E. coli*-primed and Ova peptide-pulsed B cells resulted in a significant inhibition of T cell proliferation at a T cell-B cell ratio of 1:1. In contrast, the co-incubation of T cells with *B. vulgatus*-primed B cells did not result in decreased proliferation. Only after increasing the T cell-B cell ratio to 1:5, the low immunogenic bacterium possessed T cell proliferation-preventing effects. The cultivation of T cells with naïve unstimulated B cells led to an intense T cell proliferation and a polarization toward Th1 and TH17 cells. In contrast, T cells incubated with *E. coli*-primed B cells favored a polarization shifted toward Th2 cells and Tregs. *B. vulgatus*-primed B cells also polarized T cells in a Th2 and Treg direction but simultaneously induced Th1 and Th17 cells leading to a more pro-inflammatory Th1/Th2/Th17/Treg balance (121–123). The cause for the strong T cell activation, proliferation and polarization by naïve unstimulated B cells could be the basic expression levels of MHC-II and co-stimulatory proteins in the absence of anti-inflammatory cytokines and surface molecules, which were upregulated in *E. coli*-primed B cells, as shown in previous experiments (124). In addition, after induction of Tregs via *E. coli*-primed Bregs, these two regulatory cell populations could cooperate to generate an IL-10-driven feedback loop to initiate their reciprocal activation and consequently increase their cell emergence (8, 125, 126).

The inflammation-suppressive role of Breg subsets in autoimmune disease has been demonstrated previously in *in vivo* mice models for IBD, MS, T1D, and RA, primarily achieved by the adoptive transfer before or during inflammation of *ex vivo*-activated B cells in wild type, B cell-depleted (anti-CD20 mAb treatment), or B cell-deficient (μMT) mice (10, 28, 47, 127–132). Consequently, the ability of Bregs to contribute to the control of the immune response during inflammation development and disease progression is evidently. Nevertheless, the influence of the host commensal microbiota on the induction of this regulatory cell phenotype needs to be investigated in more detail.

In earlier studies, we could demonstrate the effect of different immunogenic commensals on the differentiation and maturation of certain immune cells (e.g., CD11c^+^ DCs), concluding that a potent TLR activation provided by strong immunogenic bacteria leads to enhanced immune responses, aggravating the course of disease (60, 61, 66, 68, 69, 133, 134). Thereby, our and other groups used genetically susceptible colitis mouse models (Rag1-, IL-2-, and IL-10-deficient) exhibiting a dysregulated immune system to mimic disease development in an immune suppressed host (60, 61, 135). In detail, B cell immunity is inoperative, either due to the lack of mature B cells (Rag1^−/−^), the disturbed proliferation and induction of Bregs (IL-2^−/−^), or the dysfunction of Bregs (IL-10^−/−^) in all three colitis mouse models. Hence in these colitis models, a potent immune-stimulatory signal mediated by strong immunogenic *E. coli* exacerbated inflammation, since important counter-regulation mechanisms (such as the induction of Bregs) for the compensation of overshooting immune responses malfunctioning. Similar observation could be made with the bacterium *Helicobacter hepaticus. H. hepaticus* is a member of the mouse microbiota colonizing the lower intestine and activating innate immunity via Toll-like receptors without inducing immune pathology in a healthy host (136). *H. hepaticus* thus induces an anti-inflammatory immune response through the activation of regulatory macrophages to maintain immune homeostasis (137). However, in immune-deficient IL-10^−/−^ or Rag2^−/−^ mice, *H. hepaticus* triggered exacerbated intestinal inflammation as a result of aberrant regulatory T cell function (138–140). In conclusion, strong immunogenic bacteria, which are benignant commensals in a healthy host, provoke an uncontrolled activation of the immune system in hosts with a dysfunctional immune response, leading to inflammatory processes and the exacerbation of disease severity.

However it has already been published in mouse models for IBD and T1D that strong immunogenic bacteria, such as *E. coli* Nissle or *Helicobacter pylori* have inflammation-suppressive properties and can even prevent the onset of disease, on the condition that the host provides a functional immune system (141–143). In our study, we wanted to demonstrate the specific impact of commensal bacteria featuring different immunogenic properties on the activation of the immune system and the development of inflammation while deciphering the crucial role of regulatory B cells in these processes. Therefore, we colonized DSS-administered germfree wild type mice providing a full-featured immune system (no genetic susceptibility) to induce intestinal inflammation.

The comparison of germfree, low immunogenic *B. vulgatus*- or strong immunogenic *E. coli-*associated mice emphasizes the importance of a potent immune stimulus in DSS-induced inflammation since the colonization with *E. coli* significantly prevented intense weight loss, alleviated disease symptoms and reduced inflammation in the affected tissue. In contrast, colonization with *B. vulgatus* alleviated disease symptoms slightly, but not significantly, compared to germfree mice. Reason for that could be the weak immunogenic properties of *B. vulgatus* still provoking a low activation of B cells, minor induction of Bregs and modest inhibition of T cell proliferation and polarization, as observed in *in vitro* experiments. In addition, other bacteria-associated host immune modulating effects which are B cell- and T cell-independent, such as the restoration and education of the immature immune system and mucosal barrier present in germfree mice could be causal for the attenuated inflammation development shown in *B. vulgatus*-colonized mice. However, these potential immunogenic-independent regulatory mechanisms may be insufficient to significantly reduce inflammation processes in DSS-induced colitis. Though, these findings could explain the non-significant differences between *B. vulgatus*- and *E. coli*-colonized mice in some read outs (144, 145).

Moreover, we provide evidence that the induction of Bregs directly correlated with the severity of colonic inflammation and therefore negatively correlated with the immunogenicity of the colonizing bacteria. These findings are in line with other studies reporting a decrease of intestinal regulatory B cells in colonic inflammation in mice and humans (146–148). In line with our *in vitro* experiments, the colonization of germfree mice with *E. coli* prior to DSS-administration, and the accompanying induction of Bregs, resulted in a significantly enhanced development of Tregs and inhibition of a Th1 and Th17 polarization (Figure 7). Even though *B. vulgatus*-colonization resulted in a slightly and non-significant increased induction of Bregs, the differentiation of T helper cells is not altered in comparison to germfree mice. In contrast, the significant enhanced induction of Bregs *in vitro* and *in vivo* in *E. coli*-associated mice leading to an anti-inflammatory balance of Th1/Th2/Th17/Treg cells, marked by a pronounced differentiation of Tregs, which is decisive for the development of inflammation and consequently the possible reason for the extenuated inflammation in *E. coli*-colonized and DSS treated mice (121–123).

Thus, the increased accumulation of Bregs and Tregs established an immune-homoeostatic state *in vivo*. In combination, these two crucial regulatory cell populations might develop a powerful anti-inflammatory milieu capable of suppressing inflammation in various autoimmune diseases (149).

In conclusion, Bregs are an important interface in microbiota-driven immune regulation. They contribute to maintenance of immune homeostasis in a healthy host and counter-act emerging inflammatory processes in immune-compromised hosts via the production of suppressive molecules and the interaction with other immune cell populations. The induction and longevity of Bregs specifically depend and correlate with the strength of TLR-ligation and subsequent cell activation, provided by commensal antigens. The presented results directly link different characteristics of commensal bacteria with the immune response of the host and thus provide new insights in the inter-kingdom communication between commensals and their hosts.

## Data Availability Statement

All datasets generated for this study are included in the article/Supplementary Material.

## Ethics Statement

This study was carried out in accordance with the principles of the Basel Declaration. Protocols and experiments involving mice were reviewed and approved by the responsible Institutional Review Committee and the local authorities within H1/15, H1/17, §4 09.01.2015, §4 14.06.2017.

## Author Contributions

JM and J-SF conceived and designed the experiments. JM, CT, AL, RP, LM, AS, HY, and H-CL performed the experiments. JM, CT, and J-SF analyzed the data. JM, AL, and J-SF wrote the manuscript. All authors gave final approval to publish the article.

### Conflict of Interest

The authors declare that the research was conducted in the absence of any commercial or financial relationships that could be construed as a potential conflict of interest.

## Supplementary Material

Supplementary Figure 1 Determination of colony forming units (CFU) of germfree mice after colonization with *B. vulgatus* or *E. coli*. Feces of every mouse were collected under germ free conditions before and after colonization with bacteria and DSS-administration and were plated timely to determine the CFU.

Supplementary Figure 2 Representative flow cytometry plots of B10 cells induced by *E. coli* stimulation. Naive B cells were stimulated with *E. coli* at MOI 1 for 0, 24, 48, and 72 h. Doublets wee excluded via FSC-A/FSC-H gating and death cells were excluded by fixable viability dye. B10 cells were defined as CD19^+^CD5^+^CD1d^+^IL10^+^.

Supplementary Figure 3 Representative flow cytometry plots of T2-MZP B cells induced by *E. coli* stimulation. Naive B cells were stimulated with *E. coli* at MOI 1 for 0, 24, 48, and 72 h. Doublets were excluded via FSC-A/FSC-H gating and death cells were excluded by fixable viability dye. T2-MZP B cells were defined as CD19^+^CD21^+^CD23^+^CD24^+^.

Supplementary Figure 4 Representative flow cytometry plots of Tim-1^+^ B cells induced by *E. coli* stimulation. Naive B cells were stimulated with *E. coli* at MOI 1 for 0, 24, 48, and 72 h. Doublets were excluded via FSC-A/FSC-H gating and death cells were excluded by fixable viability dye. Tim-1^+^ B cells were defined as CD19^+^ Tim-1^+^.

Supplementary Figure 5 Analysis of Bregs in germfree mice after colonization with *B. vulgatus* or *E. coli* prior DSS administration. Changes in Bregs amount of germfree, *B. vulgatus* or *E.coli*-colonized mice 2 weeks after colonization and 2 weeks prior DSS-administration measured via flow cytometry. ^*^*p* < 0.05, ^**^*p* < 0.01, ^***^*p* < 0.001.

Supplementary Figure 6 Concentration of secreted cytokine IL-6 by stimulated B cells measured via ELISA. Naïve B cells were stimulated with *B. vulgatus (B.v.)* or *E. coli (E.c.)* at MOI 1 and PBS as control (Mock) for various time points (0, 24, 48, and 72 h) (*n* = 4). ^*^*p* < 0.05, ^**^*p* < 0.01.
