# Supplementary figures and images for: Bacterial Immunogenicity Is Critical for the Induction of Regulatory B Cells in Suppressing Inflammatory Immune Responses
Source: Front Immunol. 2020 Jan 24;10:3093. doi: 10.3389/fimmu.2019.03093 (PMC6993086; doi:10.3389/fimmu.2019.03093)

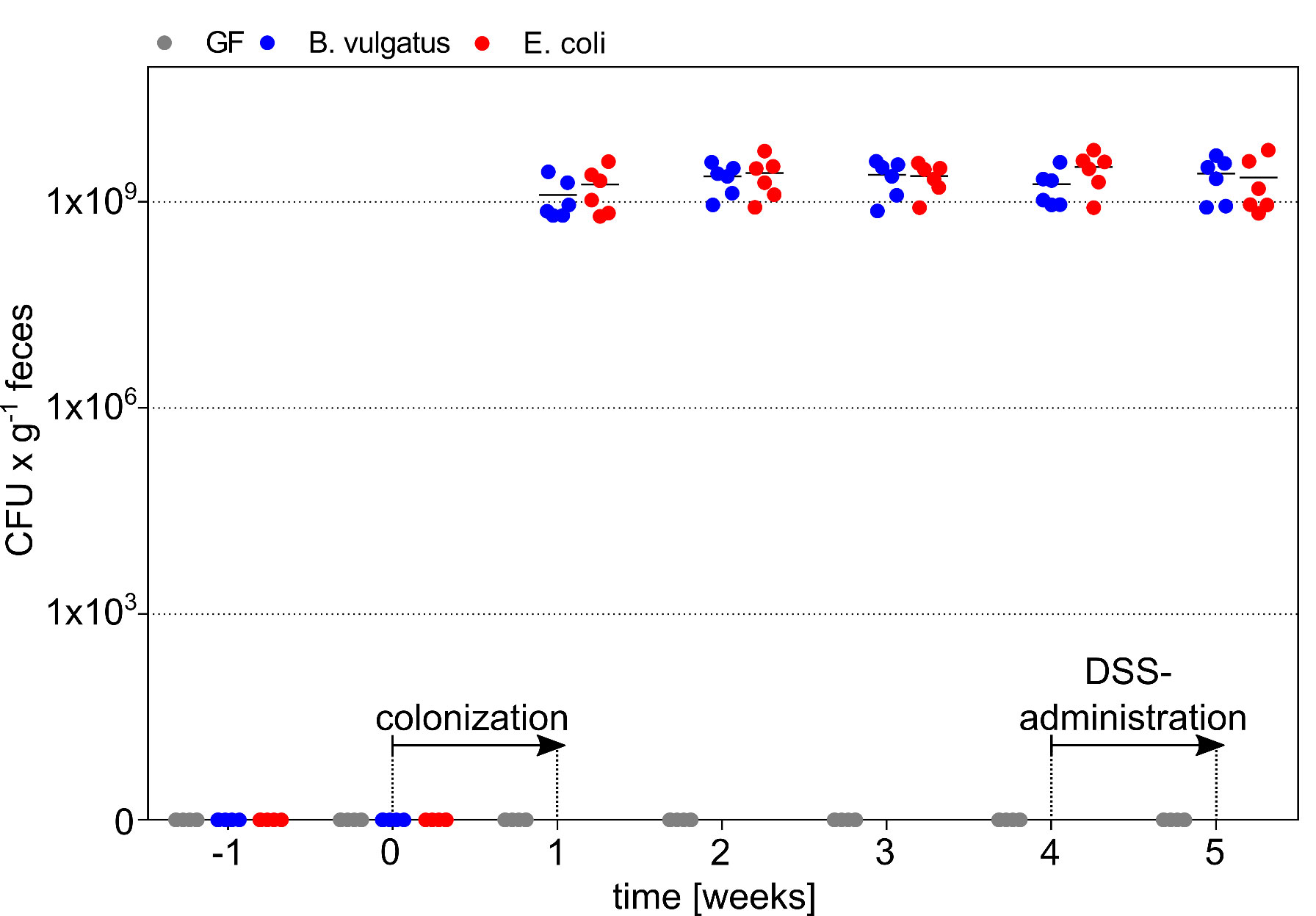

Supplement: Supplementary Figure 1 — Determination of colony forming units (CFU) of germfree mice after colonization with B. vulgatus or E. coli. Feces of every mouse were collected under germ free conditions before and after colonization with bacteria and DSS-administration and were plated timely to determine the CFU. [file Image_1.JPEG]

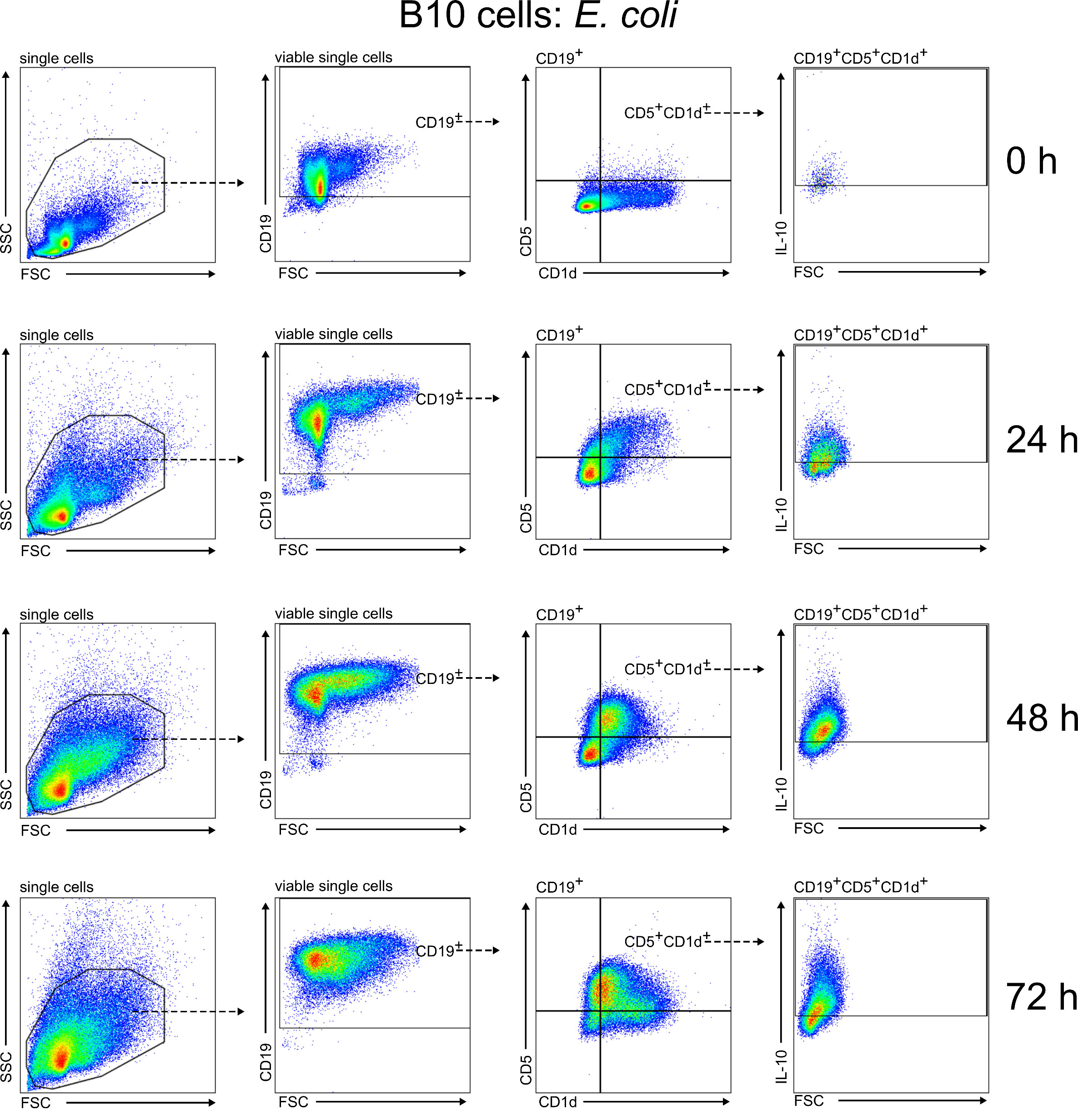

Supplement: Supplementary Figure 2 — Representative flow cytometry plots of B10 cells induced by E. coli stimulation. Naive B cells were stimulated with E. coli at MOI 1 for 0, 24, 48, and 72 h. Doublets wee excluded via FSC-A/FSC-H gating and death cells were excluded by fixable viability dye. B10 cells were defined as CD19+CD5+CD1d+IL10+. [file Image_2.JPEG]

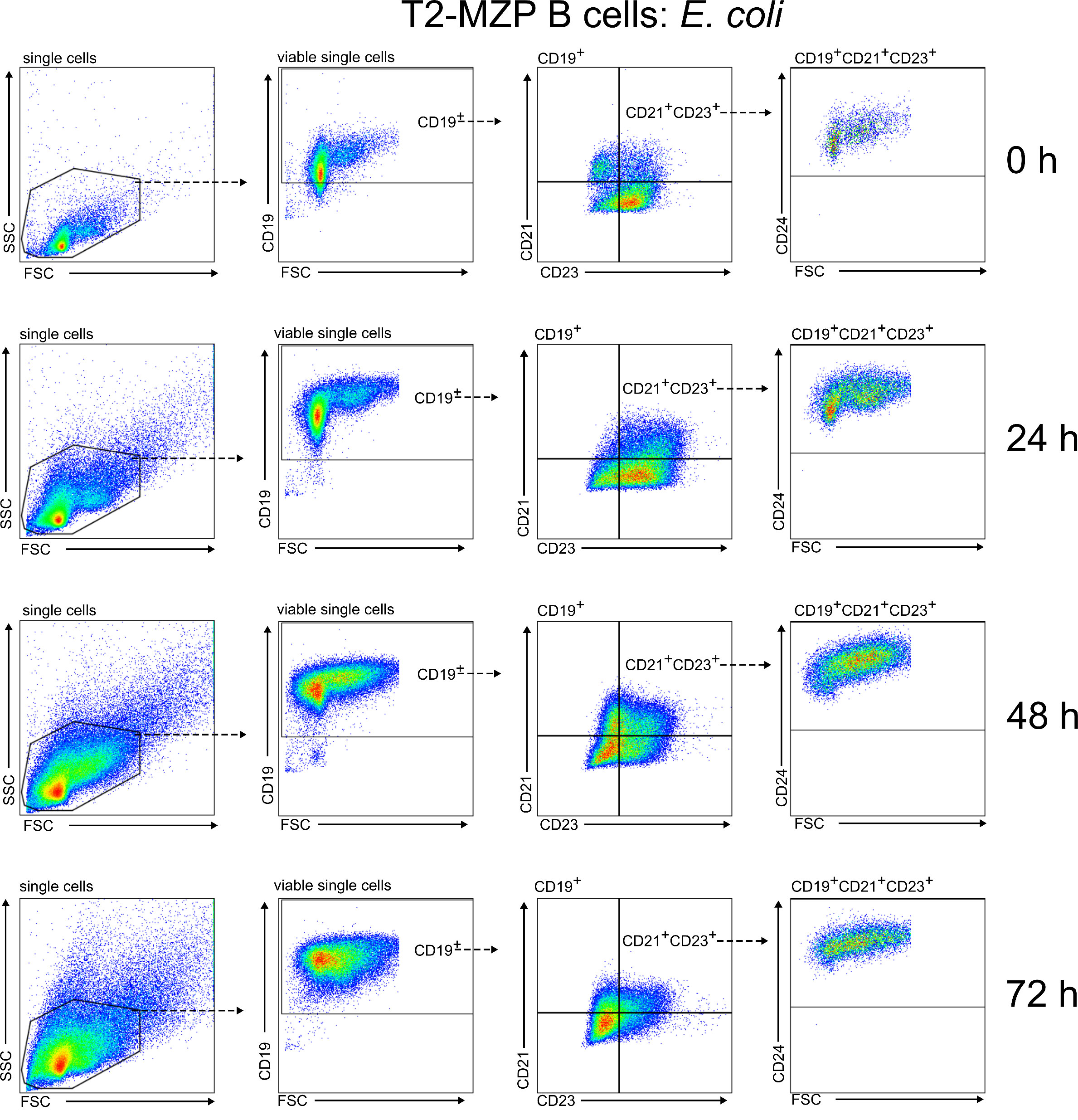

Supplement: Supplementary Figure 3 — Representative flow cytometry plots of T2-MZP B cells induced by E. coli stimulation. Naive B cells were stimulated with E. coli at MOI 1 for 0, 24, 48, and 72 h. Doublets were excluded via FSC-A/FSC-H gating and death cells were excluded by fixable viability dye. T2-MZP B cells were defined as CD19+CD21+CD23+CD24+. [file Image_3.JPEG]

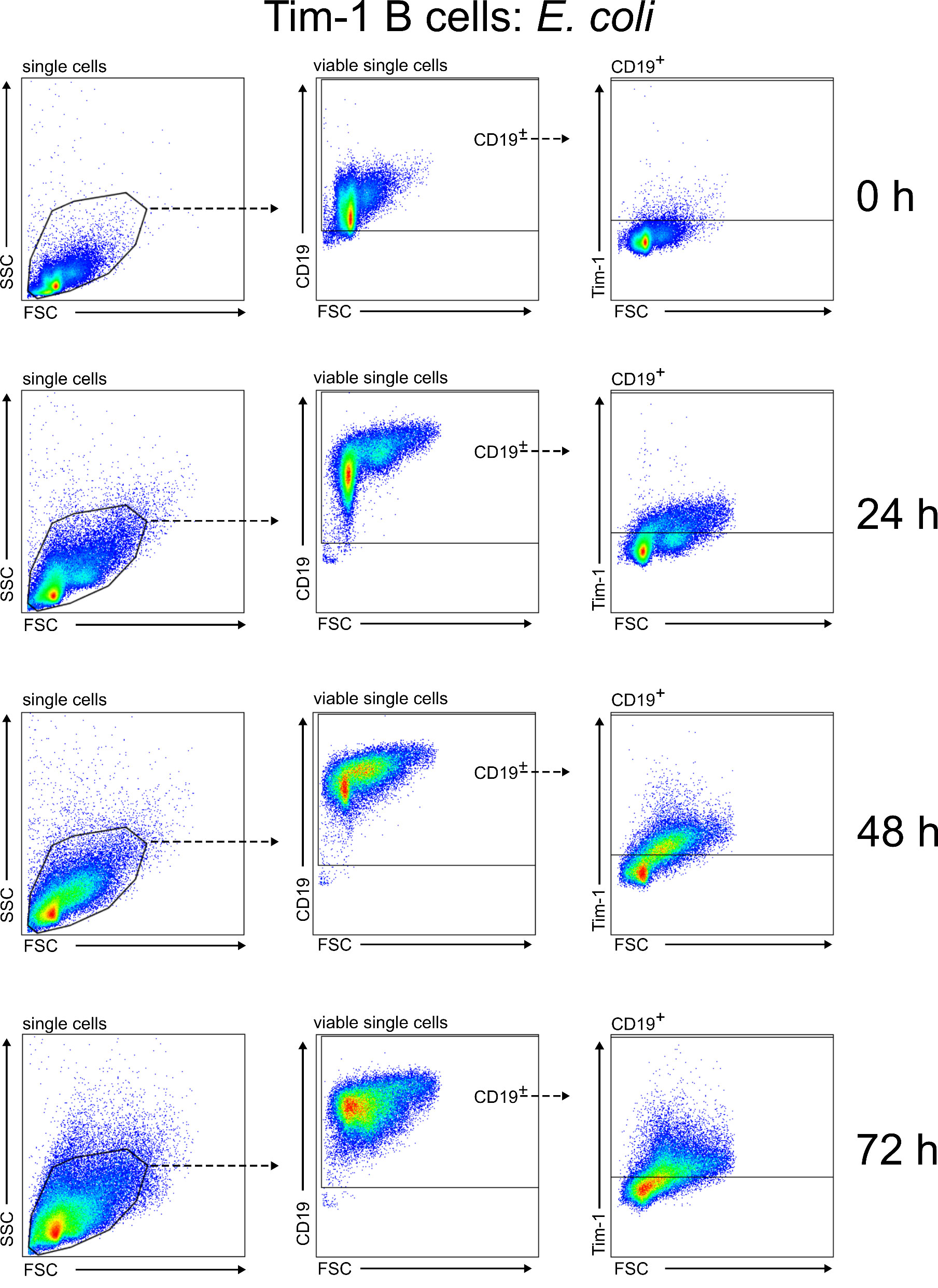

Supplement: Supplementary Figure 4 — Representative flow cytometry plots of Tim-1+ B cells induced by E. coli stimulation. Naive B cells were stimulated with E. coli at MOI 1 for 0, 24, 48, and 72 h. Doublets were excluded via FSC-A/FSC-H gating and death cells were excluded by fixable viability dye. Tim-1+ B cells were defined as CD19+ Tim-1+. [file Image_4.JPEG]

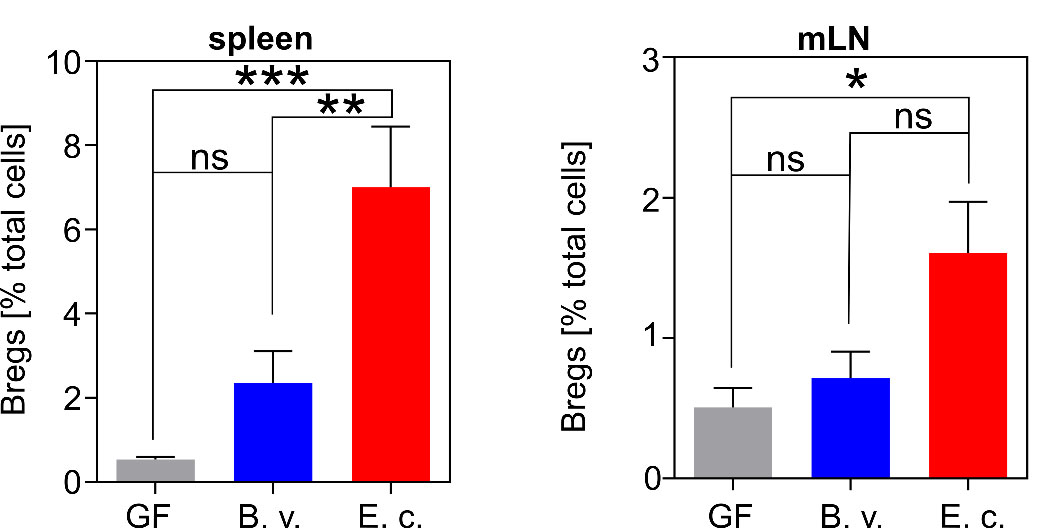

Supplement: Supplementary Figure 5 — Analysis of Bregs in germfree mice after colonization with B. vulgatus or E. coli prior DSS administration. Changes in Bregs amount of germfree, B. vulgatus or E.coli-colonized mice 2 weeks after colonization and 2 weeks prior DSS-administration measured via flow cytometry. *p < 0.05, **p < 0.01, ***p < 0.001. [file Image_5.JPEG]

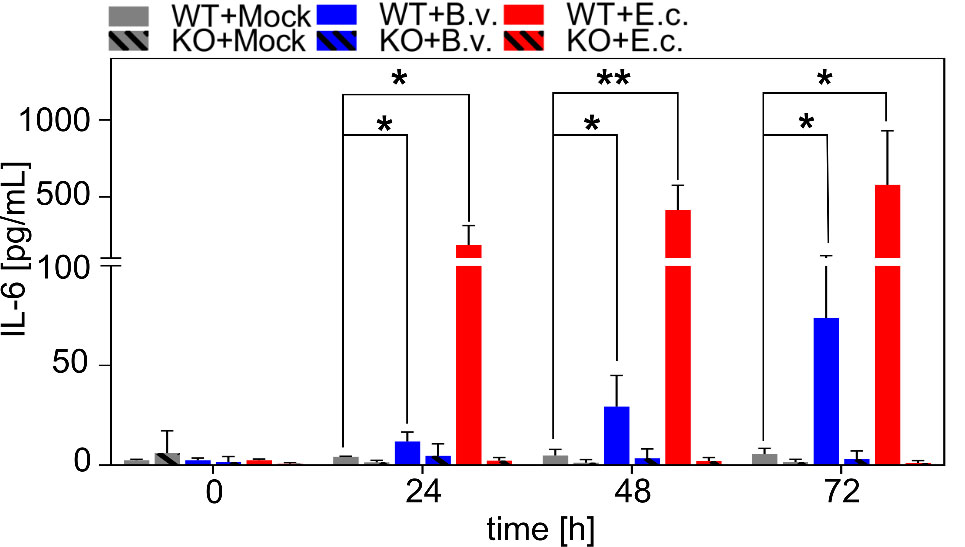

Supplement: Supplementary Figure 6 — Concentration of secreted cytokine IL-6 by stimulated B cells measured via ELISA. Naïve B cells were stimulated with B. vulgatus (B.v.) or E. coli (E.c.) at MOI 1 and PBS as control (Mock) for various time points (0, 24, 48, and 72 h) (n = 4). *p < 0.05, **p < 0.01. [file Image_6.JPEG]
